# Beyond Myelination: Possible Roles of the Immune Proteasome in Oligodendroglial Homeostasis and Dysfunction

**DOI:** 10.3389/fnins.2022.867357

**Published:** 2022-05-09

**Authors:** Miguel M. Madeira, Zachary Hage, Stella E. Tsirka

**Affiliations:** ^1^Program in Molecular and Cellular Pharmacology, Renaissance School of Medicine at Stony Brook University, Stony Brook, NY, United States; ^2^Department of Pharmacological Sciences, Renaissance School of Medicine at Stony Brook University, Stony Brook, NY, United States; ^3^Scholars in Biomedical Sciences Program, Renaissance School of Medicine at Stony Brook University, Stony Brook, NY, United States

**Keywords:** oligodendroglia, immune oligodendrocytes, multiple sclerosis, major depressive disorder, neuroinflammation

## Abstract

Oligodendroglia play a critical role in CNS homeostasis by myelinating neuronal axons in their mature stages. Dysfunction in this lineage occurs when early stage OPCs are not able to differentiate to replace dying Mature Myelinating Oligodendrocytes. Many hypotheses exist as to why de- and hypo-myelinating disorders and diseases occur. In this review, we present data to show that oligodendroglia can adopt components of the immune proteasome under inflammatory conditions. The works reviewed further reflect that these immune-component expressing oligodendroglia can in fact function as antigen presenting cells, phagocytosing foreign entities and presenting them via MHC II to activate CD4+ T cells. Additionally, we hypothesize, based on the limited literature, that the adoption of immune components by oligodendroglia may contribute to their stalled differentiation in the context of these disorders and diseases. The present review will underline: (1) Mechanisms of neuroinflammation in diseases associated with Immune Oligodendroglia; (2) the first associations between the immune proteasome and oligodendroglia and the subtle distinctions between these works; (3) the suggested functionality of these cells as it is described by current literature; and (4) the hypothesized consequences on metabolism. In doing so we aim to shed light on this fairly under-explored cell type in hopes that study of their functionality may lead to further mechanistic understanding of hypo- and de-myelinating neuroinflammatory disorders and diseases.

## Introduction

Oligodendroglia (OLN) are one of the two macroglial cell types in the central nervous system (CNS). Originating from the Neural Stem Cell (NSC) Niche as Oligodendrocyte Progenitor Cells (OPCs), OLN continuously populate the CNS in a process known as oligodendrogenesis. The lineage can be broken down into four main stages at which OLN express characteristic markers: OPCs (PDGFRα, NG2), Committed OPCs (C-OPCs; NG2, O4), Pre-Myelinating Oligodendrocytes (Pre-OL; O4, CNP), and Mature Myelinating Oligodendrocytes (M-OL; CNP, MBP).

It is in their mature stage (M-OL) that OLN carry out their most notable function of supporting neurons by wrapping axons to help propagate action potentials. Although the process of myelination is intricate, the differentiation and maturation of OLN is a complex process of its own (Emery, 2010). In mice, OPCs continually give rise to M-OL until around 4 weeks of age, at which point, oligodendrogenesis begins to slow down (Rivers et al., 2008; Psachoulia et al., 2009). The formed M-OL are then terminally differentiated cells, that are long-lived, and only around one cell per 300 turns over annually in humans (Yeung et al., 2014). However, any insult or damage to M-OL would increase the need for oligodendrogenesis and OPC differentiation, meaning a feedback loop to OPCs must exist.

Indeed, OPCs have been shown to constantly survey the environment, migrating short distances, and swiftly respond to decreases in M-OL numbers with differentiation (Hughes et al., 2013). T his process is tightly regulated by factors that both inhibit and promote differentiation. Platelet-derived growth factor (PDGF) and Fibroblast growth factor-2 (FGF-2), for example, stimulate proliferation of OPCs (Yao et al., 2017); thus, stimulation by just these factors would promote symmetric division of the cells, preventing differentiation (unless other factors are present). Triiodothyronine (T3), on the other hand, blocks OPC proliferation, inducing asymmetrical division and differentiation (Dugas et al., 2012). As these cells encounter distinct physiological scenarios and environment throughout adulthood, a balance between these and other factors maintains homeostatic oligodendrogenesis and myelination.

However, in certain disorders and diseases, demyelination occurs, suggesting that the homeostatic balance is perturbed. In Multiple Sclerosis (MS), for example, it has been shown that following initial demyelination, OPCs migrate to the site of injury and are able to differentiate to M-OLs (Menn et al., 2006). After subsequent bouts of demyelination insult, however, OPCs fail to differentiate and thus remyelination does not occur. The regulation of the many genes and factors that control these processes have been extensively studied, and many hypotheses exist as to why OPCs fail to differentiate (Rodriguez, 1989; Frohman et al., 2005).

Nonetheless, in most published works OLN are considered passive players in these processes, merely responding to changes in environmental cues. However, newer studies suggest that OLN may actually play an active role in de- and hypo-myelinating diseases and disorders, through the induction of the immune proteasome. Although evidence for this was first reported in the 1980s, little interest was placed on the topic and little to no functional research was pursued. These immune component-expressing OLN have recently been termed Immune Oligodendrocytes (ImOL) (Jäkel et al., 2019) and initial characterization suggests that their appearance may be linked to neuroinflammation.

The immune proteasome is a collection of proteins that play an important role in immune function. Together, these proteins govern processes such as phagocytosis, antigen processing, and antigen presentation, along with others (Ferrington and Gregerson, 2012). Although a higher basal expression of the immune proteasome is seen in most immune cells (especially antigen presenting cells, APCs), the upregulation of pro-inflammatory cytokines can increase its expression in all nucleated cells (Wong et al., 1984; Hirayama et al., 1986; Suzumura et al., 1986b). An important distinction must be made here between self- and non-self-presenting components of the immune proteasome. While all nucleated cells express immune proteasome components in the form of Major Histocompatibility Complex (MHC) class I molecules (serving for presenting of self-antigen) (Alberts, 2015), MHC class II molecules (serving for presenting of non-self-antigen) are typically reserved for APCs, cells with phagocytic capability that can take in and process non-self-antigen. In this review we will highlight the unique ability of OLN, under inflammatory conditions, to utilize both self and non-self components of the immune proteasome.

Here, we provide a comprehensive review of ImOL. We discuss: (1) Mechanisms of neuroinflammation in diseases associated with ImOL; (2) the first associations between the immune proteasome and OLN and the subtle distinctions between these works; and (3) the suggested functionality of these cells as it is described by current literature. With neuroinflammation as a common denominator, rodent, human, and *in vitro* studies ranging across several diseases and preclinical models will be presented. Data suggesting that oligodendrocytes do not express immune proteasome components will also be discussed. In doing so, we intend to deliver an inclusive view into the novelty of this cell type.

## Neuroinflammation: The Common Denominator

### Multiple Sclerosis

Multiple Sclerosis is an autoimmune neuroinflammatory disease characterized by demyelination in the CNS. Demyelination disrupts electrical transmission across neuronal axons and eventually leads to neurodegeneration and cell death (McDonald and Sears, 1969; Suzuki et al., 1969). As a disease in which axonal transmission is hindered, the most common symptoms affect movement, vision and cognition.

Multiple Sclerosis is well-characterized as a neuroinflammatory disease with microglia, the resident innate immune cells of the CNS, being significant mediators of inflammation (Dheen et al., 2007). Microglial ablation via CSF1R inhibition was found to attenuate experimental autoimmune encephalomyelitis (EAE), a mouse MS model which presents with activation of the peripheral immune system, demyelination and neurodegeneration (Nissen et al., 2018). Baranzini et al. (2000) reported transcriptional upregulation of interleukin 6 (IL-6) and its receptor. Chabas et al. (2001) found changes in the expression of the proinflammatory cytokine osteopontin in the spinal cord. Osteopontin^–^/^–^ mice secreted less Interferon-γ (IFN-γ) but produced higher levels of the anti-inflammatory cytokine interleukin 10 (IL-10) and displayed attenuated EAE symptoms compared to WT mice. Other important inflammatory cytokines implicated in EAE pathogenesis are interleukin 17 (IL-17), produced by Th17 cells, and IFN-γ. IL-17^–^/^–^ mice experienced mild EAE symptoms compared to WT mice, and IL-17 neutralizing antibody treatment provided partial symptom relief (Komiyama et al., 2006). IFN-γ, despite being a known proinflammatory cytokine, has a more controversial role in the development of EAE. For example, rats with chronic relapsing EAE showed decreased IFN-γ at time of disease onset, but increased IFN-γ during relapse (Tanuma et al., 1999). On the contrary, both peripheral and intrathecal administration of IFN-γ ameliorated EAE progression, whereas animals lacking IFN-γ signaling (either through anti-IFN-γ antibodies or through genetic means) appear to show increased susceptibility to EAE (Voorthuis et al., 1990; Ferber et al., 1996; Heremans et al., 1996; Furlan et al., 2001).

### Major Depressive Disorder

Major Depressive Disorder (MDD) is a psychiatric disease characterized by some or all of the following symptoms: disordered appetite, disrupted sleep patterns, general feelings of despair, loss of motivation/reward and suicidal actions or ideations. MDD is a leading cause of disability, affecting approximately 350 million people any given year (Ferrari et al., 2013). There is a subset of patients that display another clinical phenotype: low-grade, chronic inflammation.

Like in MS, M1-like microglia act as primary drivers of CNS inflammation in depression. Post-mortem brain samples from victims of suicide revealed increased microglial activation (Torres-Platas et al., 2014). It is mentioned that it is unlikely that the significant microglial activation observed occurred post-mortem, since Dibaj et al. (2010) reported long-lasting, but continuously decreasing microglial reactivity in mice for up to 10 h post-mortem. Pace et al. (2006) reported increased NF-κB DNA-binding as well as increased IL-6 in otherwise-healthy male patients, a finding that independently correlated with the severity of major depression. Several other studies have also found increased cerebrospinal fluid (CSF) levels of IL-6 in MDD patients and suicide attempters (Lindqvist et al., 2009; Sasayama et al., 2013).

MDD patients display a significant reduction in myelin content as compared to healthy controls (Sacchet and Gotlib, 2017). The neuroinflammatory environment is consistent with other diseases characterized by the loss of white matter myelin (albeit to a lesser extent), including MS. The fact that depression is a common psychological symptom of MS serves to underscore neuroinflammation as an important common characteristic of these diseases. In the context of MS, Falcão et al. (2018) found that OPCs are capable of expressing MHCII and associated genes (Falcão et al., 2018). Considering these factors holistically, it is not only possible, but likely that OPCs may also do so in the context of MDD. However, to the best of our knowledge, there is no direct evidence for this.

## Oligodendroglia: Adoption of Immune Phenotypes

An early report about the discovery of immune component-expressing OLN was made in 1981 (Ting et al., 1981). In that particular study, brain sections of untreated mice were stained with a triple-layered immunoperoxidase technique to reveal Ia^+^ (Immune response-associated) cells. Although no confirmation was given with specific OLN markers, the cells were designated as OLs based on their morphology and anatomical localization.

In the ten years following, sixteen additional publications referred to immune expression in OLN (according to our PubMed search) (Wong et al., 1984; Suzumura and Silberberg, 1985; Hirayama et al., 1986; Suzumura et al., 1986a,^1988^; Calder et al., 1988; Mauerhoff et al., 1988; Grenier et al., 1989; Lee and Raine, 1989; Sasaki et al., 1989; Kim, 1990; Ruijs et al., 1990; Styren et al., 1990; De Groot et al., 1991; Satoh et al., 1991; Turnley et al., 1991). With such publications came conflicting data on the matter. To explore these differences, it is important first make a distinction between the two main MHC classes. MHC class I and II both play a critical role in adaptive immunity by presenting fragments of protein to T cells. MHC class I proteins are present on the surface of nucleated cells and function in self-presentation to cytotoxic CD8^+^ T cells. On the other hand, MHC Class II proteins are reserved for so-called antigen-presenting cells (dendritic cells, macrophages, microglia) that present non-self peptides to CD4^+^ T helper cells (Alberts, 2015).

Of the seventeen total publications, two reported expression of MHC class II on OLN (mouse and human) (Ting et al., 1981; Kim, 1990), eleven reported MHC class I expression on OLN (mouse and human) (Wong et al., 1984; Suzumura and Silberberg, 1985; Hirayama et al., 1986; Suzumura et al., 1986a,b; Mauerhoff et al., 1988; Grenier et al., 1989; Kim, 1990; Ruijs et al., 1990; Satoh et al., 1991; Turnley et al., 1991), and thirteen specifically reported no MHC class II expression on OLN (mouse, rat, human) (Wong et al., 1984; Hirayama et al., 1986; Suzumura et al., 1986b; Calder et al., 1988; Mauerhoff et al., 1988; Grenier et al., 1989; Lee and Raine, 1989; Sasaki et al., 1989; Styren et al., 1990; De Groot et al., 1991; Satoh et al., 1991; Turnley et al., 1991; Gobin et al., 2001). Though these publications were presented in the context of different models of disease, a common denominator between them was neuroinflammation (discussed more in depth in the next section), and more specifically, IFN-γ. IFN-γ is believed to be a crucial inflammatory player in de- and hypo-myelinating diseases and disorders, and thus its effect on OLN is vital to understanding pathology (Baerwald et al., 2000). In line with this, while it is known that all nucleated cells may self-present via MHC I, the findings that IFN-γ induced MHC I expression provided an initial suggestion that oligodendroglia may not play a passive role in this group of diseases (Wong et al., 1984; Hirayama et al., 1986; Suzumura et al., 1986b).

Over the following thirty years (1992-2022), sixteen more articles were published concerning the matter. Of these, eight investigated the expression of MHC I (Horwitz et al., 1999; Baerwald et al., 2000; Redwine et al., 2001; Hoftberger et al., 2004; Na et al., 2008; Pan et al., 2020; González-Alvarado et al., 2022) and eight investigated the expression of MHC II on OLNs (Bergsteindottir et al., 1992; Horwitz et al., 1999; Yin et al., 2008; Itoh et al., 2009; Falcão et al., 2018; Jäkel et al., 2019; Nagy et al., 2020; Pan et al., 2020). The mechanistic and functional aspects reported in these publications will be further discussed in section 4 of the review.

Though the discovery of the induction of MHC I was novel and warranted further investigation, it can be argued that the finding of MHC II on OLN was, and still is, revolutionary. As more literature is discussed here, it will become clear that our understanding of the functionality of MHC I on OLN has increased drastically. However, forty-one years removed from the initial discovery, the literature still does not present a conceivable hypothesis as to why MHC II is present on OLN. Though it is clear from multiple studies that the immune phenotype is associated with inflammatory processes, specific studies on the mechanism by which OLN MHC II is expressed or induced has not yet been published (Bergsteindottir et al., 1992; Horwitz et al., 1999; Yin et al., 2008; Itoh et al., 2009; Falcão et al., 2018; Jäkel et al., 2019; Nagy et al., 2020; Pan et al., 2020). Interestingly, and in contradiction to other works, Bergsteindottir et al. (1992) reported that IFN-γ alone was not enough to induce MHC II in OLN, but that instead a combinational treatment with dexamethasone and IFN-γ was necessary (Bergsteindottir et al., 1992). The glucocorticoid dexamethasone, along with other glucocorticoids, binds to glucocorticoid receptors and is traditionally viewed as a potent repressor of inflammation and MHC expression (Schwiebert et al., 1995; Pan et al., 2001). However, the activation of glucocorticoid receptors on OLN has been shown to induce differentiation and myelinogenesis by upregulation of Proteolipid protein (PLP) (Bergsteindottir et al., 1992) which is necessary for myelin generation and deposition. In addition, the concentration of dexamethasone used in the study [10^–8^ M] was within range of free corticosteroid concentration in the brain (Sapolsky et al., 1985; Feldman, 1989). This may suggest that to express MHC II, OLN may need the presence of baseline levels of corticosteroid released from the adrenal cortex. Expanding this hypothesis further: PLP is traditionally expressed in later stage oligodendrocytes (Pre-OL, M-OL); given what was shown by Bergsteindottir et al. (1992), it is conceivable that some differentiation is necessary for MHC II expression. As we delve into more recent publications, we will again address at what stage of the OLN lineage MHC II expression is more likely to occur.

Recently during the single-cell sequencing era, the concept of Immune oligodendrocytes has been brought up in several publications that we will discuss (Falcão et al., 2018; Jäkel et al., 2019; Nagy et al., 2020). Importantly, although long after the initial discovery, Jäkel et al. (2019) and Kokkosis et al. (2022) first coined the term **Immune Oligodendrocyte**. Along with a second publication from the same research group, the sequencing data provided revealed extraordinary information about these cells (Falcão et al., 2018). In the 2018 publication, Falcão et al. (2018) performed single cell transcriptomics on spinal cords of mice induced with EAE. To their surprise, OLN not only expressed MHC I and II, but a myriad of other associated proteins and immune genes. The MHC I associated genes, *Tap1, Psmb9, B2m, H2k1, H2d1*, and *H2t23* were found to be upregulated in the EAE groups as compared to the control groups, confirming previous findings. Additionally, an RNAscope *in situ* hybridization assay was used to confirm expression of *B2m* and *Psmb9* in control and EAE spinal cord sections, showing the same results. The MHC II associated genes, *Cd74, Ctss, H2aa, H2ab1, H2eb1*, and *H2dma* were also found to be upregulated in EAE tissues. The expression of *Cd74* was confirmed via RNAscope *in situ* hybridization in EAE tissue, and the expression of MHC II was then verified in both EAE and post-mortem human MS biopsies. Importantly, these experiments confirm the presence of the functional units associated with both MHCs, potentially indicating that these cells may function as antigen-presenting cells (APCs).

Using *in vitro* co-culture experiments with naïve CD140a+ (PDGFRα) OPCs and CD45+ microglia/macrophages isolated from the spinal cord of EAE adult mice, Falcão et al. (2018) showed that the OPCs in the co-culture expressed MHC II. Lastly, in agreement with Bergsteindottir et al. (1992), the treatment of naïve CD140a+ OPCs with IFN-γ and dexamethasone induced the expression of MHC II and CD74, confirmed by both immunocytochemistry and RNAscope. However, unlike Bergsteindottir et al. (1992) the expression was also seen in the IFN-γ only treatment groups. The discrepancies in the findings of the two papers could be due to several reasons: For one, the two experiments were conducted on different species [mouse for Falcão et al. (2018) and rat for Bergsteindottir et al. (1992)]. In addition, the cells were cultured in different basal media, which could account for some difference in their differentiation potential. Besides these small differences, the findings not only confirm previous investigations, but also set the stage for a new era of discoveries in OLN biology.

Following the Falcão et al. (2018) publication, the ImOL cell type was further sequenced and investigated by Jäkel et al. (2019). In this publication, single nucleus RNA sequencing was conducted on white matter (WM) regions of post-mortem human MS patients and healthy controls. The ImOL phenotype was again found to be present, expressing many immune genes including *Cd74*. Interestingly, however, when the expression data were split by condition, it was clear that the WM of non-diseased controls also contained a population of ImOL, albeit smaller. This finding introduces an interesting new concept - potentially indicating that whatever function they serve, ImOL are necessary to maintain homeostasis. Alternatively, it could also represent a baseline level of inflammation in the study’s non-diseased controls. In addition to ImOL, eight distinct OLN clusters were found. A pseudotime analysis of the OLN lineage showed that the ImOL fell between the C-OPC stage and the Pre-OL stage. Importantly, this result may support the idea that some degree of OLN differentiation may be necessary for the adoption of immune phenotypes.

More recently the presence of ImOL was reported in MDD. In the 2020 publication, Nagy et al. (2020) conducted single nucleus RNA sequencing on post-mortem Pre-Frontal Cortex samples of 17 MDD (suicide) and 17 healthy controls (natural/accident). An integration with the Jäkel dataset (Jäkel et al., 2019) revealed that one specific OLN cluster from the Nagy study had the greatest correlation with Jäkel’s ImOL. Although the correlation was not high, the Nagy cluster was quite large and given the smaller percentage of OLN that ImOL represent, further parsing of the cells within the given cluster may yield a higher correlation. Nonetheless, the presence of these cells in MDD suggests that a lower level of inflammation may also be enough to induce the phenotype.

Together, the publications discussed in this section show that OLN may innately possess immune phenotypes under homeostatic conditions, and that inflammatory triggers may increase the numbers of ImOL. Although there is a strong correlation between inflammatory processes and the presence of this novel cell type, causational work has not been done yet. Thus, the insights on mechanistic immune function of ImOL are limited. In the next section we attempt to explore the limited literature that is available and hypothesize some potential functionality for these cells.

## Mechanistic Insights: Functionality of Immune Oligodendrocytes

The innate and adaptive immune systems work intricately with one another to maintain homeostasis. APCs and T cells play central roles in these processes. Via MHC II, APCs will typically present non-self antigens to CD4+ T helper (T_*h*_) cells. These activated T_*h*_ cells will in turn generate memory T_*h*_ cells and activate T cytotoxic (T_*c*_) cells. Activated T_*c*_ cells can then create memory T_*c*_ cells as well as destroy infected cells (Lefrançois and Obar, 2010). This entire process has been well mapped when it comes to APCs; however, the ability of MHC II expressing OLN to induce CD4+ T_*h*_ cells has just recently become a subject of interest.

Based on our theoretical knowledge of the major histocompatibility complexes, phagocytosis of foreign pathogens by APCs is necessary for MHC II presentation. In support of OLN functioning as APCs, they have been shown to phagocytose (Falcão et al., 2018; Kirby et al., 2019). Falcão et al. (2018) treated OPC cultures with 1 μm fluorescent microspheres for 24 h. Up to 48% of the OPCs in the culture were able to uptake several microspheres. Cytochalasin D, an inhibitor of phagocytosis, was successfully used to reduce the phagocytosis of the microspheres, confirming an active phagocytosis process, as opposed to passive diffusion of the microspheres. Surprisingly, treatment with IFN-γ had no effect on the phagocytic capabilities of the OPCs. However, if we attempt to connect these experiments with the acquisition of immune phenotypes by OLN there are several questions left unanswered, and these experiments do not truly evaluate the phagocytic capabilities of ImOL. For one, the experiments which showed the induction of MHC II with IFN-γ *in vitro* were conducted for 72 h, making these 24 h experiments too short to induce ImOL formation. Since the ImOL phenotype was shown to lie between the C-OPC and Pre-OL stages [Jäkel et al. (2019) and Nagy et al. (2020)], evaluating solely at the OPC stage does not fully capture the phagocytic capabilities of ImOL. This leaves us with the question: If the ImOL phenotype was first induced for 72 h, and then microspheres were added to culture, would ImOL show a higher uptake than non-immune expressing OLN?

To address some of these questions Falcão et al. (2018) conducted experiments in a more disease-specific context. They evaluated whether OPCs could phagocytose pHrodo-labeled myelin. Expectedly, the OPCs were able to phagocytose myelin. Although still lacking the evidence for Tap1 processing and the presentation to CD4+ T_*h*_ cells, this experiment could substantiate a hypothesis that OLN play an active role in their own destruction. The authors assessed the effects of 72 h co-culture of OPCs with CD4+ T cells isolated from 2D2 mice which contain T cells that express the T cell receptor for the myelin oligodendrocyte glycoprotein (MOG 35-55) peptide. The OPCs were either untreated, or treated with IFN-γ and/or MOG and were co-cultured with either naïve, memory, or effector T cells. The co-culturing of memory T cells with OPCs pretreated with IFN-γ and MOG increased their survival compared to untreated OPCs, suggesting a MHC II-mediated effect. Both naïve and memory CD4+ T cells increased their proliferation when OPCs were exposed to MOG before the co-culture, suggesting again an MHC II-dependent effect. Importantly, the pre-treatment of OPCs with IFN-γ did not affect the proliferation of T cells. However, subsequent experiments showed that the presence of MOG pre-treated OPCs induced IFN-γ secretion from CD4+ memory T cells, indicating that the exogenous IFN-γ treatment may have been redundant.

Together the results from Falcão et al. (2018) indicate that OLN possess the ability to phagocytose and present non-self-antigen to CD4+ T cells, inducing their proliferation. In theory, these CD4+ cells would then activate CD8+ cells, which would in turn kill OLN as the target cell. This feedback loop fits in perfectly in the context of hypo- and de-myelinating disorders. Indeed, another piece of this puzzle has been shown by Kirby et al. (2019). In this publication, using mouse models of MS and *in vitro* experiments, it is shown that via MHC I presentation OPCs can directly activate CD8+ T cells and subsequently become their target.

Although the work presented in this section has been conducted in the context of MS, it is likely that the same concepts may apply to other neuroinflammatory disorders, including hypo-myelinating depression. It is apparent from this collection of works that ImOL do not only express immune components but are able to use them; in doing so, they play an active role in hypo- and de-myelinating pathogenesis, as well as a potential role in homeostatic maintenance.

## Adoption of Immune Proteasome and Hypothesized Effects on Metabolism

Cellular metabolism is the process by which cells govern and fulfill biological functions. Unsurprisingly, this network of biochemical reactions is inextricably linked to energy. Whether it is its production or consumption, all cells must properly allocate energy to perform their given functions. This means that a given cell cannot perform all functions at 100% all the time, but must pick which processes are most important. The adoption of the immune proteasome by OLN provides an interesting, yet ill-illuminated, example of this: What metabolic functions are ImOL losing to harbor and use such a great extent of the immune proteasome?

Other observations suggest that the self-induced killing of OPCs is not the sole culprit in hypo- and de-myelinating disorders. It has been shown that in addition to OPC death, OPCs in MS may also simply fail to differentiate to M-OLs. Myelination is a crucial evolutionary event in the development of higher vertebrates (Tepavcevic, 2021), and thus the process of oligodendrogenesis consumes considerable amounts of energy. Once at the M-OL stage, production of myelin requires lipid and protein syntheses that are energetically very costly (Tepavcevic, 2021). Like most cells in the brain, OPCs at homeostasis utilize glucose and oxidative phosphorylation to generate ATP and energy (Mayorga-Weber et al., 2022). To produce myelin, however, they also metabolize lactate and generate lipids. OLN glucose is oxidized through the pentose phosphate pathway to promote NADPH production and lipid biosynthesis (Sanchez-Abarca et al., 2001). They do not have the high basal glycolytic activity that microglia and astrocytes exhibit (Meyer and Rinholm, 2021), but they have the potential to induce high levels glycolysis when needed. In addition, once myelin has been produced and axons are wrapped, the support provided to neurons requires constant shuttling of metabolites – further energy investment (Tepavcevic, 2021).

As these differentiating OLN cells are deciding whether to push to differentiation or remain in ImOL stage, a decrease in energy, a shift in energy sources and pathways activated, and the pro-inflammatory environment could result in the induction of Integrated Stress Response (ISR) proteins (Pakos-Zebrucka et al., 2016). Indeed, the expression of ISR proteins has been shown to increase in OLN under stress conditions (Pavitt, 2005; Gow and Wrabetz, 2009; Clayton et al., 2017). In an in culture approach it was recently shown that activation of the ISR resulted in decreased remyelination in a cuprizone model of chemical demyelination (Roth et al., 2021), while Chen et al. reported that prolongation of the ISR in combination with exposure to IFN-γ significantly enhanced remyelination by increasing the number of remyelinating oligodendrocytes and myelinated axons (Chen et al., 2021). Changes in energy sources, coupled with changes in global protein synthesis, may explain why ImOL are practically non-existent at the M-OL stage. For the time being the ISR and ImOL connection remains correlational; however, it presents a reasonable hypothesis for the induction of ImOL and should be further investigated.

The opposite, however, may also be true. Once the ImOL phenotype has been adopted and the immune proteasome is in use, there may not be enough energy for the cells to then differentiate. Although this hypothesis is not substantiated by specific data, it is entirely possible that lack of energy is the reason for stalled differentiation. This would mean that not only are OLN self-targeting by adopting immune phenotypes but also committing to remaining at an earlier stage in the lineage, further contributing to overall loss of white matter.

## Concluding Remarks

Oligodendroglia play key roles in the CNS by not only myelinating neuronal axons but also providing them with trophic support (Wilkins et al., 2001; Dai et al., 2003). Given the importance of M-OL-mediated myelin wrapping in accelerating action potentials, the OLN lineage has evolved an interconnected feedback loop between its early and late stages (Menn et al., 2006). As mature OL die off, signaling to OPCs (either from the dying M-OL, or from the cellular microenvironment around them) will induce OPC proliferation and differentiation so that they eventually ‘fill in’ the needs in myelination. However, the existence of de- and hypo-myelinating disorders and diseases ultimately points toward dysfunction in the lineage, as one of the contributors to the lack of myelin repair to the initial normal myelination levels. Although many hypotheses exist as to why demyelination may occur, here we reviewed literature that supports the adoption of the immune proteasome by OLN and suggests that it plays a role in white matter loss.

Neuroinflammation is critical for the pathophysiology of MS, MDD, and other diseases and disorders of the CNS. Chief among the similarities is the upregulation of pro-inflammatory molecules including IFN-γ, IL-6, and NF-κB. In addition, although not as severe as in MS, the loss of white matter is also seen in MDD. In this review, we presented evidence that inflammation can induce the expression of immune components in OLN. Further, from more recent works, it has been shown that these components are functional and OLN seem to double as APCs when necessary. By presenting myelin antigen to CD4+ T cells, ImOL induce their own death, contributing to demyelination. Some works also showed that ImOL are present in control samples. Although this could indicate that their function is necessary to maintain homeostasis, it could also be indicative of some baseline inflammation in control patients.

Importantly, we must make note that functional studies involving other (not T cells) immune cells, in the context of ImOL, are limited. However, we hypothesize here that the innate immune cells of the CNS, microglia, do play a role in the loss of white matter and induction of ImOL – whether it be through their secretion of pro-inflammatory cytokines or phagocytosis of OLN. Additionally, given the involvement of T helper cells shown here, it is conceivable that the humoral immunity component of the adaptive immune system, B cells, may also play a role in hypo- and de-myelinating disorders. This may not only be by secretion of pro-inflammatory factors but generation of abnormal immunoglobulins against OLN. Indeed, several reports have implicated B cell dysfunction in the context of MS and MDD (Ahmetspahic et al., 2018; Gregson et al., 2019; Cencioni et al., 2021).

Moreover, it seems from literature that ImOL appear between the C-OPC and Pre-OL stage. Although we hypothesize here that induction of the immune proteasome could lead to stalled differentiation and further contribute to white matter loss, more work must be conducted to shed light on the mechanisms by which ImOL affect the pathophysiology of de- and hypo-myelinating disorders and diseases.

**FIGURE 1:**
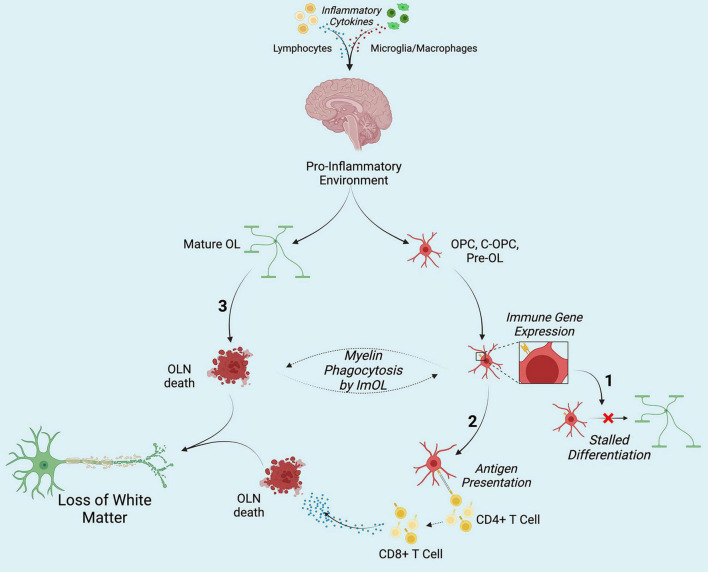
Hypothesized contribution of ImOL to hypo- and de-myelinating disorders and diseases. Secretion of pro-inflammatory cytokines from lymphocytes (and possibly from microglia macrophages) results in the adoption of the immune proteasome by oligodendroglia. (1) Once OLN become ImOL, a host of factors including energy deficits stalls differentiation. (2) Presentation of non-self (MBP, MOG) and self-antigens by ImOL to CD4+ T cells results in the activation of CD8+ T cells which then induce OLN death. (3) Cytokines directly affect Mature OL and cause their death. Together, in no particular order, the three contribute to loss of white matter structures by stopping early stage OLN from differentiation, targeting mature OL (M-OL) and inducing their death, and priming CD8+ T cells toward myelin. Created with Biorender.com.

Together, the works compiled here represent an interesting and ill-lit subject. Though first presented in 1981, not much interested seemed to be placed in the concept of Immune component-expressing OLN. As the sequencing era has emerged, the literature has seen a resurgence in this topic, providing ample evidence for their existence and functionality. Further mechanistic exploration of ImOL is necessary to truly characterize this novel cell type, and future works should remain cognizant of the fact that this exploration could open new avenues in understanding and treated neuroinflammatory disorders and diseases (Figure 1).

## Author Contributions

MM, ZH, and ST structured, edited, and wrote the sections. All authors contributed to the article and approved the submitted version.

## Conflict of Interest

The authors declare that the research was conducted in the absence of any commercial or financial relationships that could be construed as a potential conflict of interest.

## Publisher’s Note

All claims expressed in this article are solely those of the authors and do not necessarily represent those of their affiliated organizations, or those of the publisher, the editors and the reviewers. Any product that may be evaluated in this article, or claim that may be made by its manufacturer, is not guaranteed or endorsed by the publisher.
